# Diagnosis of postpartum depression and associated factors in South Africa: a cohort study of 47,697 women

**DOI:** 10.1017/S2045796025100103

**Published:** 2025-07-29

**Authors:** C. Gastaldon, V. Whitesell Skrivankova, G. Schoretsanitis, N. Folb, K. Taghavi, M. A. Davies, M. Cornell, G. Salanti, C. Mesa Vieira, M. Tlali, G. Maartens, M. Egger, A. D. Haas

**Affiliations:** 1 Institute of Social and Preventive Medicine, Faculty of Medicine, University of Bern, Bern, Switzerland; 2 Department of Psychiatry, Psychotherapy and Psychosomatics, Hospital of Psychiatry, University of Zurich, Zurich, Switzerland; 3 Department of Psychiatry, The Zucker Hillside Hospital, Northwell Health, Glen Oaks, NY, USA; 4 Department of Psychiatry, Zucker School of Medicine at Northwell/Hofstra, Hempstead, NY, USA; 5 Medscheme, Cape Town, South Africa; 6 International Agency for Research on Cancer, Lyon, France; 7 School of Public Health, Centre for Infectious Disease Epidemiology & Research, University of Cape Town, Cape Town, South Africa; 8 Division of Clinical Pharmacology, Department of Medicine, University of Cape Town, Cape Town, South Africa; 9 Population Health Sciences, Bristol Medical School, University of Bristol, Bristol, UK

**Keywords:** depression, epidemiology, mood disorders postpartum, risk factors, women

## Abstract

**Aims:**

About one-third of South African women have clinically significant symptoms of postpartum depression (PPD). Several socio-demographic risk factors for PPD exist, but data on medical and obstetric risk factors remain scarce for low- and middle-income countries and particularly in sub-Saharan Africa. We aimed to estimate the proportion of women with PPD and investigate socio-demographic, medical and obstetric risk factors for PPD among women receiving private medical care in South Africa (SA).

**Methods:**

In this longitudinal cohort study, we analysed reimbursement claims from beneficiaries of an SA medical insurance scheme who delivered a child between 2011 and 2020. PPD was defined as a new International Classification of Diseases, 10th Revision diagnosis of depression within 365 days postpartum. We estimated the frequency of women with a diagnosis of PPD. We explored several medical and obstetric risk factors for PPD, including pre-existing conditions, such as HIV and polycystic ovary syndrome, and conditions diagnosed during pregnancy and labour, such as gestational diabetes, pre-term delivery and postpartum haemorrhage. Using a multivariable modified Poisson model, we estimated adjusted risk ratios (aRRs) and 95% confidence intervals (CIs) for factors associated with PPD.

**Results:**

Of the 47,697 participants, 2,380 (5.0%) were diagnosed with PPD. The cumulative incidence of PPD increased from 0.8% (95% CI 0.7–0.9) at 6 weeks to 5.5% (5.3–5.7) at 12 months postpartum. PPD risk was higher in individuals with history of depression (aRR 3.47, 95% CI [3.14–3.85]), preterm delivery (1.47 [1.30–1.66]), PCOS (1.37 [1.09–1.72]), hyperemesis gravidarum (1.32 [1.11–1.57]), gestational hypertension (1.30 [1.03–1.66]) and postpartum haemorrhage (1.29 [0.91–1.85]). Endometriosis, HIV, gestational diabetes, foetal stress, perineal laceration, elective or emergency C-section and preeclampsia were not associated with a higher risk of PPD.

**Conclusions:**

The PPD diagnosis rate was lower than anticipated, based on the PPD prevalence of previous studies, indicating a potential diagnostic gap in SA’s private sector. Identified risk factors could inform targeted PPD screening strategies.

## Introduction

The postpartum phase is a high-risk period for women’s mental health, as they experience social, psychological and hormonal changes. One of the most common complications of these changes is the onset of postpartum depression (PPD), which may occur up to 1 year after delivery (Bennett *et al.*, 2004).

The global prevalence of women with clinically significant symptoms of PPD has been estimated at 17.4% (95% confidence interval [CI] 16.7–18.2), with large variations across countries and settings (Flynn *et al.*, 2004; O’Hara and McCabe, 2013; Wang *et al.*, 2021). Women in low- and middle-income countries (LMICs) exhibit higher rates of clinically significant PPD symptoms, estimated at 19.0% (15.5–23.0) through validated screening tools (Gelaye *et al.*, 2016; Roddy Mitchell *et al.*, 2023). Recent systematic reviews and meta-analyses pooled rates of 16.8%–24.3% in sub-Saharan Africa (Nweke *et al.*, 2022; Roddy Mitchell *et al.*, 2023)and 31.0%–38.8% in South Africa (SA; Gelaye *et al.*, 2016; Wang *et al.*, 2021). To date, individual studies conducted in SA had small sample sizes (between 67 and 1035 women) and mainly screened women with validated tools for clinically significant PPD symptoms, showing large variability of estimates, ranging from 8.8% to 53.0% (Duma and Madiba, 2020; Govender *et al.*, 2020; Hung *et al.*, 2014; Mokwena and Masike, 2020; Peltzer *et al.*, 2018; Pingo *et al.*, 2017; Ramchandani *et al.*, 2009). Such variability may be due to heterogeneity in settings and characteristics of included women, which could limit the comparability of different studies.

PPD may have implications for maternal and offspring well-being and health (Netsi *et al.*, 2018; Roddy Mitchell *et al.*, 2023; Srinivasan *et al.*, 2020; Wisner *et al.*, 2013), including alterations of maternal physical and psychological health, as well as infant physical health, and motor, cognitive, emotional and behavioural development (Slomian *et al.*, 2019). Timely detection and management of PPD could improve depressive symptoms and their consequences (Dennis and Dowswell, 2013), yet both need improvement in many LMICs (Fonseca *et al.*, 2020). In recent years, there has been an increasing interest in risk factors for PPD, which could aid in identifying at-risk women who should be the target of screening programmes (Dennis and Dowswell, 2013). Predictors of PPD identified in both LMICs and high-income countries (HICs) include maternal age, lower education, unwanted pregnancy, intimate partner violence or other forms of violence, poor family or partner support, low income, unemployment, exposure to war or disasters, HIV and a history of depression (Gastaldon *et al.*, 2022; Hutchens and Kearney, 2020; Roddy Mitchell *et al.*, 2023; Zhang *et al.*, 2019). In contrast, obstetric and medical predictors of PPD have been mainly studied in HICs (Hutchens and Kearney, 2020), with evidence linking PPD with gynaecological conditions predating the pregnancy, such as premenstrual syndrome (Cao *et al.*, 2020) or polycystic ovary syndrome (Schoretsanitis *et al.*, 2022), pregnancy complications and obstetric complications such as gestational diabetes (Arafa *et al.*, 2019), preeclampsia (Caropreso *et al.*, 2020). Caesarean section (C-section) (Xu *et al.*, 2017), preterm delivery (de Paula Eduardo *et al.*, 2019) and postpartum haemorrhage (Schoretsanitis *et al.*, 2024). However, these predictors have barely been investigated in cohorts from LMICs (Gastaldon *et al.*, 2022; Roddy Mitchell *et al.*, 2023; Schoretsanitis *et al.*, 2022, 2024).

Given the high PPD prevalence in SA estimated in previous studies through ad hoc research screenings, it is essential to estimate the proportion of women who are diagnosed with PPD under routine clinical practice circumstances, as PPD might be unrecognized and untreated in everyday clinical practice. Additionally, research on PPD risk factors is needed to develop effective targeted screening and treatment strategies (Fonseca *et al.*, 2020; Gelaye *et al.*, 2016; Wang *et al.*, 2021). We aimed to estimate the proportion of women diagnosed with PPD with routinely collected insurance clinical data and investigate socio-demographic, medical and obstetric risk factors of PPD among female beneficiaries of a large South African medical insurance scheme.

## Methods

We conducted a cohort study analysing longitudinal reimbursement claims of beneficiaries of a large South African medical insurance scheme. The study included beneficiaries aged between 10 and 55 years, who were enrolled in the medical insurance scheme between 1 January 2011 and 15 March 2020 (i.e. start of COVID-19 measures in SA), delivered a new-born in that period and had health insurance coverage at least from the beginning of the pregnancy (37 weeks before delivery) and at least 6 weeks after delivery. We excluded beneficiaries with unknown sex, age or delivery date. Additionally, those who submitted a claim with depression diagnoses during pregnancy were excluded, as we focused on new episodes of depression with postpartum onset and these participants were not considered at risk for the onset of a new PPD episode in the postpartum period, considering that they were already depressed during pregnancy. We excluded these women to avoid misclassifying ongoing or chronic depression as PPD, thereby ensuring a more accurate assessment of new-onset PPD. Including women with depression during pregnancy could result in an overestimation of PPD prevalence, as it might capture women already in depressive episodes before childbirth, as reimbursement claims do not provide information about timing of remission. We included only the first delivery for beneficiaries with more than one delivery recorded in the selected timeframe (Supplementary eFigure 1).

We defined the study measures based on the International Classification of Diseases, 10th Revision (ICD-10) diagnoses from outpatient and hospital claims with hospital procedure codes (Current Procedural Terminology [CPT]) and medication claims (Anatomical Therapeutic Chemical [ATC] classification system). We defined baseline as the participants’ delivery date. Deliveries were identified based on ICD-10 codes for delivery (O80–O84), complications of labour and delivery (O60–O75) and CPT codes for other medical or surgical procedures related to delivery (Supplementary eTable 1).

We defined an episode of PPD as a new ICD-10 diagnosis of a depressive episode (F32), recurrent depressive episode (F33) or postpartum/postnatal depression (F53.0) within 365 days after delivery. We used a time window of 365 days to account for potential delays in the detection of PPD (Roddy Mitchell *et al.*, 2023).

We examined several risk factors for PPD, including conditions pre-existing the pregnancy, i.e. polycystic ovary syndrome, endometriosis and HIV (Supplementary eTable 2), conditions diagnosed during pregnancy, i.e. gestational diabetes, gestational hypertension, vitamin D deficiency and hyperemesis gravidarum, and perinatal or obstetric complications such as foetal stress or complications during labour and delivery, preterm delivery, perineal laceration, elective or emergency C-section, preeclampsia and postpartum haemorrhage. Additional risk factors considered were age at delivery (<18, 18–24, 25–29, 30–34, 35–40, ≥41 years) and a history of depression before pregnancy, defined as having at least one depression diagnosis in the 3 years before pregnancy (ICD-10 codes for risk factors are reported in Supplementary eTable 3).

Among participants diagnosed with gestational diabetes, hypertension and HIV, we distinguished between participants with ‘treated’ and ‘untreated’ conditions. Participants who received the respective treatment, i.e. antidiabetic medications, antihypertensive medications or antiretroviral medication for HIV treatment during pregnancy, were classified as ‘treated’, while those who did not receive any such medication were classified as ‘untreated’ (ATC codes are reported in Supplementary eTable 4).

We summarized the characteristics of study participants using descriptive statistics stratified by PPD status. We used the Kaplan–Meier method to estimate the cumulative incidence of PPD. Participants were followed from their delivery date to the earliest of: diagnosis of PPD, end of their insurance coverage or 15 March 2020. We used multivariable modified Poisson regression models with robust standard errors to estimate adjusted risk ratios (aRRs) and 95% CIs for risk factors of PPD (Zou, 2004). Poisson regression models allow accounting for differential follow-up time (e.g. drop-out from the insurance scheme) and interpreting estimated coefficients directly as risk ratios (RRs) rather than odds ratios (ORs), which are commonly provided based on logistic regression. Time under follow-up after delivery, truncated to 365 days, was used as exposure time. Poisson regression models included PPD risk factors, self-identified population group (Black, Mixed Ancestry, White, Indian/Asian and unknown) and calendar year.

To confirm the robustness of the main analysis, we also performed a multivariate Cox proportional hazards model, estimating adjusted hazard ratios (aHRs) and 95% CIs for factors associated with PPD, adjusted for the same factors. Furthermore, we performed Poisson regression models using a subset of participants with a minimum of 3 years of insurance coverage before pregnancy. By focusing on this subsample, we aimed to increase the likelihood of identifying any underlying history of depression.

In a secondary analysis, we examined the risk of PPD in beneficiaries with treated versus untreated PPD risk factors to explore the role of treatment in the association.

We performed statistical analyses in Stata (Version 16. College Station, TX: StataCorp).

## Results

Among people enrolled in the medical insurance scheme between 1 January 2011 and 15 March 2020, 49,983 delivered a live-born child and had insurance coverage from the start of the pregnancy until at least 6 weeks postpartum. After excluding 1,570 (3.1%) people who received a depression diagnosis during pregnancy and 446 (0.8%) with unknown age, we included 47,967 participants in our analysis. The mean age at delivery was 30.3 years (standard deviation [SD] 6.1). The medical histories indicated that 6,925 (14.4%) had received an HIV diagnosis, 2,485 (5.2%) had endometriosis, 922 (1.9%) had polycystic ovary syndrome and 2,969 (6.2%) had been diagnosed with depression before the onset of pregnancy. Documented pregnancy complications included 1,976 participants (4.1%) with a diagnosis of hyperemesis gravidarum, 1,022 (2.1%) with gestational hypertension, 1,112 (2.3%) with gestational diabetes and 14 (<0.1%) with vitamin D deficiency. Regarding complications during labour or postpartum, 20,274 participants (42.3%) had an elective C-section, while 10,100 (21.1%) required an emergency C-section. There were 4,257 preterm deliveries (8.9%) and 3,829 cases (8.1%) of foetal distress or complicated labour. Additionally, 2,863 participants (6.1%) were diagnosed with preeclampsia, 1,466 (3.1%) experienced perineal laceration, 432 (0.9%) suffered postpartum haemorrhage and 247 (0.5%) developed eclampsia (Table 1).

**Table 1.** Characteristics of women with and without postpartum depression (PPD)

	PPD	No PPD	Total
	*N* (%) = 2,380 (5.0)	*N* (%) = 45,587 (95.0)	*N* (%) = 47,967 (100.0)
**Age at delivery, years**			
10−17	40 (1.7)	921 (2.0)	961 (2.0)
18−24	259 (10.9)	6,266 (13.7)	6,525 (13.6)
25−29	706 (29.7)	12,927 (28.4)	13,633 (28.4)
30−34	793 (33.3)	14,677 (32.2)	15,470 (32.3)
35−40	488 (20.5)	9,081 (19.9)	9,569 (19.9)
41+	94 (3.9)	1,715 (3.8)	1,809 (3.8)
Mean (SD)	30.7 (6.0)	30.3 (6.1)	30.3 (6.1)
**Medical and obstetrics risk factors**			
HIV	333 (14.0)	6,592 (14.5)	6,925 (14.4)
PCOS	79 (3.3)	843 (1.8)	922 (1.9)
Endometriosis	166 (7.0)	2,319 (5.1)	2,485 (5.2)
Gestational hypertension	73 (3.1)	949 (2.1)	1,022 (2.1)
Gestational diabetes	58 (2.4)	1,054 (2.3)	1,112 (2.3)
Vitamin D deficiency	2 (0.1)	12 (0.0)	14 (0.0)
Hyperemesis gravidarum	134 (5.6)	1,842 (4.0)	1,976 (4.1)
Foetal distress/complicated labour	180 (7.6)	3,714 (8.1)	3,895 (8.1)
Preterm delivery	321 (13.5)	3,936 (8.6)	4,257 (8.9)
Perineal laceration	55 (2.3)	1,411 (3.1)	1,466 (3.1)
Elective C-section	1,072 (45.0)	19,202 (42.1)	20,274 (42.3)
Emergency C-section	523 (22.0)	9,577 (21.0)	10,100 (21.1)
Preeclampsia	167 (7.0)	2,740 (6.0)	2,907 (6.1)
Eclampsia	17 (0.7)	230 (0.5)	247 (0.5)
Postpartum haemorrhage	31 (1.3)	401 (0.9)	432 (0.9)
History of depression	468 (19.7)	2,501 (5.5)	2,969 (6.2)
**Insurance coverage before delivery, years**			
Median (IQR)	2.2 (1.3−3.5)	2.2 (1.3−3.6)	2.2 (1.3−3.6)
**Insurance coverage after delivery, years**			
Median (IQR)	2.9 (1.4−5.1)	2.5 (1.1−4.8)	2.5 (1.1−4.9)

IQR = interquartile range, PCOS = polycystic ovary syndrome, PPD = postpartum depression, SD = standard deviation.

**Figure 1.**
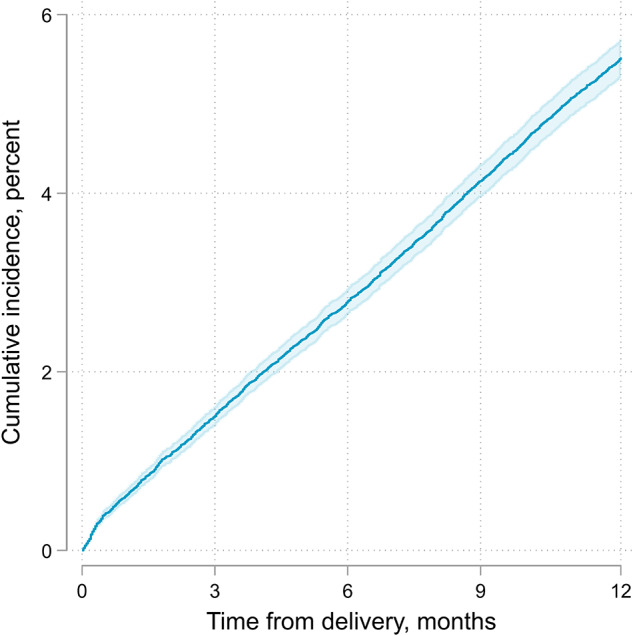
Cumulative incidence of postpartum depression diagnosis.

A total of 2,380 participants (5.0%) fulfilled PPD criteria. Among them, 2,331 (97.9%) were diagnosed with a depressive episode or disorder (F32–F33) according to ICD-10 criteria, and 81 (3.4%) were diagnosed specifically with PPD (F53). The cumulative incidence of PPD diagnoses showed a gradual increase, from 0.8% (95% CI 0.7%–0.9%) at 6 weeks postpartum, to 2.8% (95% CI 2.7%–3%) at 6 months and reaching 5.5% (95% CI 5.3%–5.7%) at 12 months postpartum (Fig. 1). The incidence of PPD diagnoses remained constant throughout the study duration. The calendar year of delivery was not associated with PPD incidence in the unadjusted (RR 1.01, 95% CI 0.99–1.03) analysis or in the analysis adjusted for age and population group (aRR 0.99, 95% CI 0.98–1.01).

**Figure 2.**
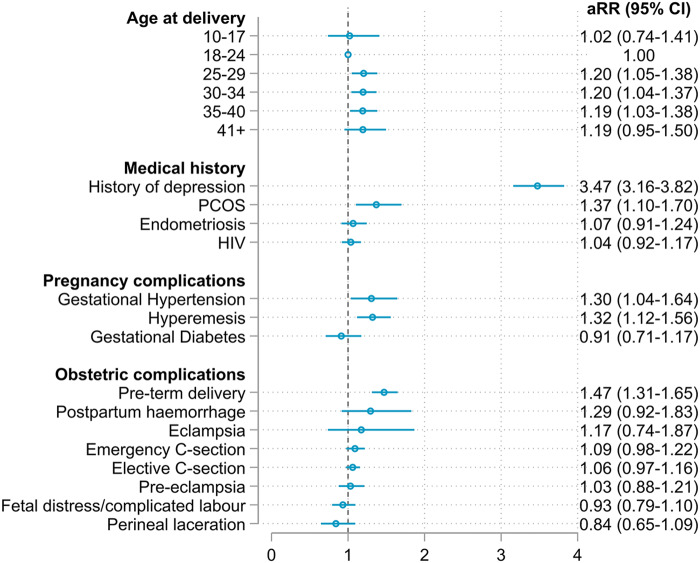
Adjusted risk ratios (aRRs) and 95% confidence intervals (95% CI) for the association between risk factors and PPD. The Poisson regression model was adjusted for all variables shown in the model, calendar year and self-identified population group. PCOS: polycystic ovary syndrome.

We presented the results (aRRs and corresponding 95% CI) of the main analysis in Fig. 2, all factors for which we explored the association with PPD are reported. Participants aged 25 years or older displayed approximately a 20% higher risk of PPD compared to participants aged 18–24 years, while adolescents aged 10–17 were at similar risk. A depression diagnosis in the 3 years before pregnancy was the strongest risk factor for PPD (aRR 3.47, 95% CI 3.14–3.85), followed by preterm delivery (aRR 1.47, 95% CI 1.30–1.66), PCOS (aRR 1.37, 95% CI 1.09–1.72), hyperemesis gravidarum (aRR 1.32, 95% CI 1.11–1.57), gestational hypertension (aRR 1.30, 95% CI 1.03–1.66) and postpartum haemorrhage (aRR 1.29, 95% CI 0.91–1.85). We found no evidence of association between PPD and the remaining hypothesized risk factors. The association between vitamin D deficiency and PPD could not be evaluated due to the small number of participants diagnosed with vitamin D deficiency in the cohort. In the multivariate Cox proportional hazards model, aHRs were similar to the results of the main analysis, confirming the main results (Supplementary eTable 5).

Table 2 shows the characteristics of the subset of 16,368 participants with at least 3 years of insurance coverage before delivery and aRRs for factors associated with PPD in this group. Consistent with the main analysis, a history of depression within the 3 years before pregnancy was strongly associated with PPD, demonstrating nearly four times higher risk in participants with a history of depression. The PPD risk related to the remaining risk factors was consistent with the main analysis. Slightly higher point estimates of aRR with overlapping 95% CI were observed for most risk factors, except for age between 25 and 29 years, endometriosis, gestational diabetes and hyperemesis gravidarum which had lower aRRs compared to the main analysis.

**Table 2.** Characteristics of participants with and without depression in the subset with at least 3 years of insurance coverage before delivery

	PPD	No PPD	Total	
	*N* (%) = 782 (4.8%)	*N* (%) = 15,586 (95.2%)	*N* (%) = 16,368 (100.0)%	aRR (95% CI)
**Age at delivery, years**				
10−17	25 (3.2)	515 (3.3)	540 (3.3)	1.29 (0.82−2.02)
18−24	80 (10.2)	2,325 (14.9)	2,405 (14.7)	1
25−29	147 (18.8)	2,961 (19.0)	3,108 (19.0)	1.13 (0.86−1.49)
30−34	271 (34.7)	4,973 (31.9)	5,244 (32.0)	1.29 (1.00−1.67)
35−40	215 (27.5)	3,911 (25.1)	4,126 (25.2)	1.30 (1.00−1.70)
41+	44 (5.6)	901 (5.8)	945 (5.8)	1.20 (0.83−1.75)
Mean (SD)	31.6 (6.8)	30.9 (7.0)	30.9 (7.0)	–
**Risk factors**				
HIV	132 (16.9)	2,611 (16.8)	2,743 (16.8)	1.04 (0.85−1.27)
PCOS	34 (4.3)	384 (2.5)	418 (2.6)	1.40 (0.99−1.99)
Endometriosis	71 (9.1)	1,091 (7.0)	1,162 (7.1)	1.03 (0.80−1.32)
Gestational hypertension	32 (4.1)	406 (2.6)	438 (2.7)	1.36 (0.95−1.96)
Gestational diabetes	21 (2.7)	409 (2.6)	430 (2.6)	0.82 (0.53−1.28)
Vitamin D deficiency	0 (0.0)	8 (0.1)	8 (0.0)	NA
Hyperemesis gravidarum	41 (5.2)	572 (3.7)	613 (3.7)	1.27 (0.93−1.74)
Foetal distress/complicated labour	66 (8.4)	1,420 (9.1)	1,487 (9.1)	0.92 (0.70−1.23)
Preterm delivery	117 (15.0)	1,506 (9.7)	1,623 (9.9)	1.47 (1.20−1.79)
Perineal laceration	21 (2.7)	516 (3.3)	537 (3.3)	0.97 (0.62−1.51)
Elective C-section	371 (47.4)	6,704 (43.0)	7,075 (43.2)	1.13 (0.97−1.33)
Emergency C-section	179 (22.9)	3,354 (21.5)	3,533 (21.6)	1.14 (0.93−1.40)
Preeclampsia	62 (7.9)	1,031 (6.6)	1,093 (6.7)	1.04 (0.79−1.36)
Eclampsia	6 (0.8)	80 (0.5)	86 (0.5)	1.19 (0.52−2.70)
Postpartum haemorrhage	13 (1.7)	157 (1.0)	170 (1.0)	1.37 (0.79−2.37)
History of depression	233 (29.8)	1,433 (9.2)	1,666 (10.2)	3.44 (2.94−4.01)
**Insurance coverage before delivery, years**				
Median (IRQ)	4.4 (3.6−5.7)	4.4 (3.6−5.7)	4.4 (3.6−5.7)	
**Insurance coverage after delivery, years**				
Median (IRQ)	2.6 (1.4−4.3)	2.2 (1.0−3.9)	2.2 (1.0−3.9)	

IQR = interquartile range, NA = not available, PCOS = polycystic ovary syndrome, PPD = postpartum depression, SD = standard deviation. All analyses are adjusted for self-identified population group.

All analyses are adjusted for self-identified population group.

Of the medical conditions evaluated – HIV, gestational diabetes and gestational hypertension – only gestational hypertension was associated with PPD. We further investigated this association with a model that separated the gestational hypertension group into treated and untreated. Participants with untreated hypertension exhibited a higher risk of PPD (aRR 1.56, 95% CI 1.18–2.06), while those with treated hypertension (aRR 1.03, 95% CI 0.71–1.49) showed a PPD risk comparable to participants without hypertension.

## Discussion

In our cohort study of nearly 50,000 South African insurance beneficiaries who delivered a child between 2011 and 2020, 5.5% were diagnosed with PPD within 12 months after delivery. The most significant risk factor for PPD was a previous diagnosis of depression in the 3 years before pregnancy. Other notable risk factors included preterm delivery, polycystic ovary syndrome, hyperemesis gravidarum, gestational hypertension and postpartum haemorrhage.

The proportion of women diagnosed with PPD in our study (5.5% at 1 year) appears to be lower than any previous estimations in SA, sub-Saharan countries, but also in other LMICs and HICs (Roddy Mitchell *et al.*, 2023; Wang *et al.*, 2021). There may be several possible explanations for this discrepancy, namely essential variability and limitations of diagnostic tools for PPD, in timing of the assessment of PPD and in settings or populations included across different studies. First, regarding the variability in diagnostic tools, to our knowledge, this is the first study estimating the proportion of women diagnosed with PPD according to routine clinical diagnostic criteria in SA’s private sector, i.e. ICD-10 codes. Consequently, it was hard to directly compare the proportion of women with PPD in our study with the prevalence of PPD estimated in other studies using different diagnostic instruments. Pooled estimates from a meta-analysis of cross-sectional studies employing the Edinburgh Postnatal Depression Scale (EPDS) for diagnosis report a 23% prevalence for LMICs and 24% for sub-Saharan countries (Roddy Mitchell *et al.*, 2023). Although the EPDS is a validated tool broadly employed in research, it has only been validated as a screening tool. Its use as a diagnostic tool may lead to overestimations of the prevalence of PPD by including women with mild or sub-clinical symptoms of depression but without a major depressive episode (Tsai *et al.*, 2013). Specifically, for scores ≥12, EPDS has a sensitivity of 74% and a specificity of 89% for PPD diagnosis, with a low positive predictive value and a high risk for false positives (Matthey *et al*., 2017). When accounting for the diagnostic inaccuracies of the EPDS, the true underlying prevalence of PPD has been estimated at 16% (Gonçalves Pacheco *et al.*, 2023). Similarly, estimates from studies conducted in sub-Saharan Africa that employed clinical diagnostic criteria similar to ours, such as the Diagnostic and Statistical Manual of Mental Illnesses (DSM), were also higher than ours. Specifically, prevalence ranged from 11% to 15% in Nigeria (Adewuya *et al.*, 2005; Uwakwe, 2003) and 26% to 33% in Zimbabwe (Chibanda *et al.*, 2010), and reached 25% within socially disadvantaged populations in SA (Lawrie *et al.*, 1998). Reasons for the lower prevalence of PPD observed in our study compared to others may relate to the inherent limitations of the claims data employed. Specifically, a PPD diagnosis in our dataset might only be recorded if treatment is sought and claimed. This could result in underreporting due to various barriers to care, including financial constraints, stigma and competing responsibilities, which might deter women from seeking or claiming treatment.

Another explanation for this discrepancy could be the timing of diagnosis in our study. PPD onset often occurs within the first 6 weeks postpartum (Gonçalves Pacheco *et al.*, 2023; Uwakwe, 2003). In our cohort, only about 1% of women were diagnosed during the first 6 weeks, suggesting that many women were diagnosed with a delay or not diagnosed at all. Both explanations – the disparity between the anticipated underlying prevalence and the findings from our study – suggest a potential diagnostic gap in SA’s private sector which should be further investigated and adequately addressed through the early identification of women at risk of PPD and the adaptation of targeted screenings for such women.

An alternative explanation could be that the true prevalence of PPD in our cohort was lower than in other sub-Saharan settings due to socio-economic differences. Our study included women from SA’s private sector, who have a higher socio-economic status compared to other population groups in sub-Saharan Africa. Given that socio-demographic factors significantly influence the development and persistence of PPD, it is possible that the actual prevalence of PPD in our study population was lower than in other sub-Saharan regions characterized by greater socio-economic disadvantages. However, it must be noted that even in HICs, where access to mental healthcare is better, PPD prevalence is higher, with a pooled estimate of 15.54 (4.9–16.2) (Wang *et al.*, 2021).

The strongest predictor of PPD was a recent history of depression before pregnancy, increasing the risk of PDD almost fourfold compared to no history of recent depression. A history of depression has been consistently identified as one of the main risk factors in previous studies and systematic reviews (Gastaldon *et al.*, 2022; Hutchens and Kearney, 2020; Nweke *et al.*, 2022). The estimated increase in PPD risk of 47% (95% CI 31%–65%) among women with preterm deliveries aligns with the literature. A meta-analysis of 12 studies found an increase in the odds of PPD of 79% associated with preterm birth, with substantial heterogeneity between studies (de Paula Eduardo *et al.*, 2019). Similarly, other pregnancy or labour complications such as gestational hypertension (Strapasson *et al.*, 2018), hyperemesis gravidarum (Austin *et al.*, 2019), postpartum haemorrhage (Liu *et al.*, 2021), emergency C-section (Xu *et al.*, 2017) or gestational diabetes (Arafa and Dong, 2019) have been shown to be associated with PPD (Colditz, 2010; Schoretsanitis and Deligiannidis, 2020; Song and Chung, 2010). Polycystic ovary syndrome, a condition usually diagnosed before conception, emerged as one of the strongest risk factors (around 40% higher risk). Of note, polycystic ovary syndrome has been associated with a higher risk of depression at any time in women’s life, including the postpartum period (Pope *et al.*, 2015; Schoretsanitis *et al.*, 2022). We did not find HIV to be an important risk factor for PPD, in contrast with a recent meta-analysis showing a 42% (95% CI 12–80) increase in the odds of PPD among women living with HIV compared to those without HIV (Zhu *et al.*, 2019). The lack of observed associations between HIV and PPD in our study could be due to higher socio-economic status, enhanced access to comprehensive HIV care, including psychosocial support, available to our private sector cohort. This group may be exposed to fewer socio-economic stressors than the majority of individuals living with HIV in sub-Saharan Africa, who are generally less advantaged in terms of resources (Peltzer *et al.*, 2018).

Another important finding of our study is that many of the identified risk factors for PPD are modifiable. Only women with untreated gestational hypertension showed an increased risk of PPD, whereas those who received treatment for gestational hypertension did not display an increase in risk. Although these findings should be interpreted with caution due to the observational nature of our study, which inherently limits the interpretation of causal relationships, they might suggest that the management of PPD risk factors emerging before or during pregnancy could potentially have a role in reducing the risk of PPD (Wang *et al.*, 2021)

Screening programmes have shown promise in enhancing early diagnosis, case finding and treatment of PPD (Waqas *et al.*, 2022). Studies in HICs have shown the feasibility and benefits of PPD screening in outpatient settings (Chaudron *et al.*, 2004; Morris-Rush *et al.*, 2003; Waqas *et al.*, 2022), yet there is no evidence for the effectiveness of these programmes in LMICs, including SA. Despite limited evidence and a lack of specific recommendations for screening in the perinatal period, South African mental health guidelines recommend routine screening for mental illness during pregnancy (Department of Health, 2013). The feasibility of implementing routine screening programmes in resource-limited settings is controversial, as it requires substantial financial and human resources to ensure follow-up and comprehensive management (Kagee *et al.*, 2013). Positive screening results require further diagnostic assessment, appropriate treatment and support (Kagee *et al.*, 2013). For these reasons, US and UK guidelines recommend implementing screening programmes only when sufficient resources are available for screening, diagnostic assessment and effective treatment (Waqas *et al.*, 2022). In SA’s private sector, where sufficient resources may be available, PPD screening might be cost-effective, potentially mitigating negative maternal and infant outcomes (Waqas *et al.*, 2022; Wilkinson *et al.*, 2017). Conversely, considering the large PPD burden and the shortage of mental healthcare professionals in SA’s public sector (Ruffieux *et al.*, 2021), large-scale screening and treatment by mental health professionals may be unrealistic (World Health Organization, 2017). The task-sharing of PPD screening and treatment with lay health workers, an approach often suggested to overcome the shortage of trained healthcare workers (Dennis and Dowswell, 2013), did not prove to be effective in treating depression in a randomized trial conducted in SA (Lund *et al.*, 2020). Considering the constraints of limited resources, targeted screening programmes for women at high PPD risk might be a viable option to allocate resources more effectively.

Our findings must be interpreted in the context of certain limitations. First, we assessed beneficiaries’ health status based on ICD-10 diagnoses derived from reimbursement claims. As a result, we might have missed women with undiagnosed depression or medical and obstetric conditions or who did not submit a claim for reimbursement. Alternative tools, such as the EPDS, are widely used as screening instruments and can be valuable in research or exploratory settings. However, they do not directly correspond to clinical diagnoses in clinical practice. Therefore, although ICD-10 codes were the only available diagnostic criteria for this cohort, it must be noted that they represent one of the most pragmatic criteria to estimate the proportion of women formally diagnosed with PPD in real-world clinical practice in the South African private sector, reflecting the vast majority of diagnoses made by clinicians linked to reimbursement claims.

Second, we could not account for subjective birth experience and psychosocial risk factors of PPD that could confound the relationship between medical and obstetric factors and PPD, such as socio-economic status, family support and level of education. Third, our study only included data from a private-sector medical insurance scheme. Thus, findings do not necessarily apply to women accessing the public healthcare sector. Women using public health services generally have lower socio-economic status and may be at a higher risk of experiencing complications during pregnancy than those who access private services. Despite these limitations, our study is one of the most extensive cohort studies conducted in LMICs investigating PPD and its medical and obstetric risk factors.

Our findings point to a substantial discrepancy between the expected prevalence of PPD and the actual diagnosis rate within SA’s private sector, especially during the early postpartum period. This discrepancy highlights a potential diagnostic gap, with numerous cases of PPD possibly remaining undiagnosed and untreated or diagnosed and treated too late. To bridge this gap, the implementation of PPD screening and streamlined treatment programmes could facilitate early diagnosis, timely intervention and appropriate treatment. Implementing targeted screening, particularly among women with a history of depression or other identified risk factors, could maximize the benefit of available resources and ensure those at the highest risk receive appropriate attention and care.

Future research could focus on the development and piloting of targeted screening and treatment programmes suitable for various settings, evaluating their effectiveness and cost-effectiveness.

## Supporting information

10.1017/S2045796025100103.sm001 Gastaldon et al. supplementary material Gastaldon et al. supplementary material

## Data Availability

Data were obtained from the International epidemiology Databases to Evaluate AIDS-Southern Africa (IeDEA-SA). For inquiries about the data, readers can contact them through the online form available at https://www.iedea-sa.org/contact-us/. Further information is available from the corresponding author upon request. The code for the statistical analysis will be made available upon reasonable request.

## Supplementary material

The supplementary material for this article can be found at https://doi.org/10.1017/S2045796025100103.
