# Supplementary material for: Diagnosis of postpartum depression and associated factors in South Africa: a cohort study of 47,697 women
Source: Epidemiol Psychiatr Sci. 2025 Jul 29;34:e41. doi: 10.1017/S2045796025100103 (PMC12450542; doi:10.1017/S2045796025100103)
Supplement: Gastaldon et al. supplementary material [file S2045796025100103sup001.docx]

**Supplementary material**

**eFigure 1: Flow diagram showing selection of eligible individuals for analysis**


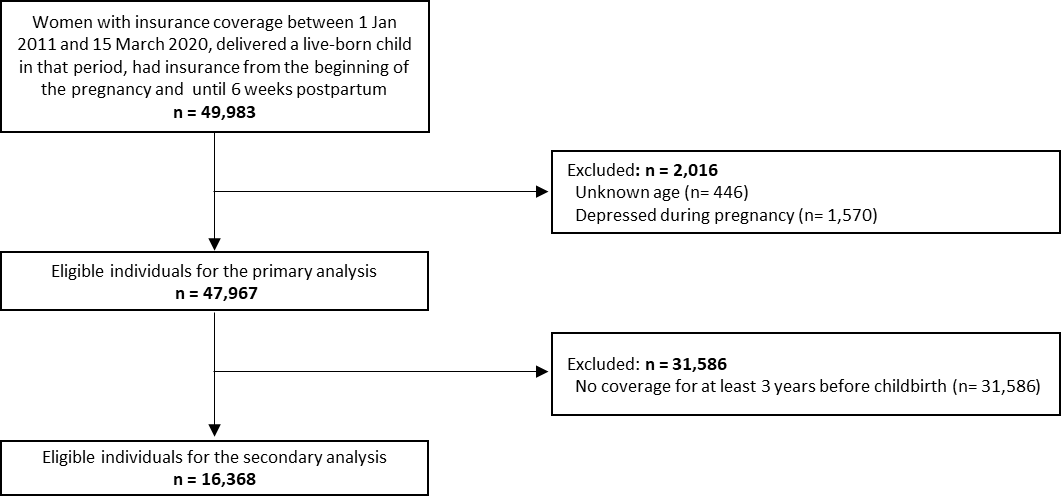


**eTable 1. List of delivery codes**

| **Code description** | **Code type** | **Code** |
| --- | --- | --- |
| Delivery |  | O80- O84 |
| Single spontaneous delivery | ICD-10 | O80 |
| Single delivery by forceps and vacuum extractor | ICD-10 | O81 |
| Single delivery by caesarean section | ICD-10 | O82 |
| Other assisted single delivery | ICD-10 | O83 |
| Multiple delivery | ICD-10 | O84 |
| Complications of labour and delivery | ICD-10 | O60-O75 |
| Failed induction of labour | ICD-10 | O61 |
| Abnormalities of forces of labour | ICD-10 | O62 |
| Long labour | ICD-10 | O63 |
| Obstructed labour due to malposition and malpresentation of fetus | ICD-10 | O64 |
| Obstructed labour due to maternal pelvic abnormality | ICD-10 | O65 |
| Other obstructed labour | ICD-10 | O66 |
| Labour and delivery complicated by intrapartum hemorrhage, not elsewhere classified | ICD-10 | O67 |
| Labour and delivery complicated by fetal stress [distress] | ICD-10 | O68 |
| Labour and delivery complicated by umbilical cord complications | ICD-10 | O69 |
| Perineal laceration during delivery | ICD-10 | O70 |
| Postpartum haemorrhage | ICD-10 | O72 |
| Retained placenta and membranes, without hemorrhage | ICD-10 | O73 |
| Complications of anesthesia during labour and delivery | ICD-10 | O74 |
| Other complications of labour and delivery, not elsewhere classified | ICD-10 | O75 |
| Other medical or surgical procedures related to delivery |  |  |
| To dilate cervix prior to delivery | CPT | 59200 |
| Procedure after childbirth | CPT | 59160 |
| Episiotomy | CPT | 59300 |
| Antepartum and labour and post-partum care for full term vaginal delivery | CPT | 59400 |
| Admission to the hospital for delivery (vaginal delivery) | CPT | 59409 |
| Antepartum and labour and post-partum care for full term vaginal delivery, episiotomy, forceps | CPT | 59410 |
| Turning the fetus in headfirst position | CPT | 59412 |
| Admission to the hospital for delivery (abdominal incision) | CPT | 59510 |
| Admission to the hospital for delivery (abdominal incision) | CPT | 59514 |
| Admission to the hospital for delivery (C- section) | CPT | 59515 |
| C-section and hysterectomy | CPT | 59525 |
| Antepartum and labour and post-partum care for full term vaginal delivery, episiotomy, forceps (previous C-section who delivered vaginally) | CPT | 59610 |
| Admission to the hospital for delivery (forceps, episiotomy) | CPT | 59612 |
| Admission to the hospital for delivery (forceps, episiotomy, vaginal delivery) | CPT | 59614 |
| Admission to the hospital for delivery (C-section after vaginal attempt) | CPT | 59618 |
| Admission to the hospital for delivery (intensive management and abdominal incision) | CPT | 59620 |
| Admission to the hospital for delivery (intensive management and abdominal incision after vaginal attempt) | CPT | 59622 |
| Anaesthesia for vaginal delivery | CPT | 01960 |
| Anaesthesia for C-section | CPT | 01961 |
| Anesthesia services for an urgent hysterectomy following delivery. | CPT | 01962 |
| Anesthesia services for a patient undergoing cesarean hysterectomy, which is removal of the uterus during a cesarean section. The provider does not provide labor analgesia or anesthesia care. | CPT | 01963 |
| Neuraxial anesthesia services for a patient undergoing a planned vaginal delivery. | CPT | 01967 |
| Anesthesia services for a patient undergoing a cesarean delivery after administration of neuraxial anesthetic. | CPT | 01968 |

**eTable 2. List of diagnoses, medications, and test results indicative of HIV**

| **Diagnoses** | **ICD10 code** |
| --- | --- |
| Human immunodeficiency virus (HIV) disease | B20-B24 |
| Asymptomatic HIV infection status | Z21 |
| Laboratory evidence of HIV | R75 |
| HIV disease complicating pregnancy, childbirth and the puerperium | O98.7 |
| **Antiretroviral medication for treating HIV** | **ATC code** |
| Protease inhibitors | J05AE |
| Nucleoside and nucleotide reverse transcriptase inhibitors | J05AF |
| Non-nucleoside reverse transcriptase inhibitors | J05AG |
| Integrase inhibitors | J05AJ |
| Antivirals for treatment of HIV infections, combinations | J05AR |
| **Antiretroviral medication used in pre- or post-exposure prophylaxis** | **ATC code** |
| Tenofovir disoproxil and emtricitabine (TDF/FTC) | J05AR03 |
| Tenofovir alafenamide (TAF) | J05AF13 |
| Emtricitabine (FTC) | J05AF09 |
| Lamivudine (3TC) | J05AF05 |
| **Laboratory test** | **Value** |
| Confirmatory HIV test | Positive |

**eTable 3. List of diagnoses of maternal complications during pregnancy**

| **Diagnoses** | **ICD10 code** |
| --- | --- |
| Medical history |  |
| History of Depression | F32, F33 |
| Endometriosis | N80 |
| PCOS | E82.2 |
| Medical complications during pregnancy |  |
| Gestational diabetes | O24, E10-14 |
| Gestational hypertension | O13, I10-I13 |
| Vitamin D deficiency | E-55 |
| Hyperemesis gravidarum | O21.0, O21.1 |
| Perinatal and obstetric complications |  |
| Fetal stress/complications during labor and delivery | O68 |
| Preterm delivery | O60.0-O60.3 |
| Perineal laceration | O70 |
| Elective C-section | O82.0 |
| Emergency C-section | O82.1 |
| Pre-eclampsia | O14 |
| Eclampsia | O15 |
| Postpartum hemorrhage | O72 |

PCOS=polycystic ovary sindrome

**eTable 4. Secondary exposure variables**

| Condition | ICD-10 code | ATC code |
| --- | --- | --- |
| Untreated gestational diabetes | O24, E10-14 | None |
| Treated gestational diabetes | O24, E10-14 | A10 |
| Untreated gestational hypertension | O13, I10-I13 | None |
| Treated gestational hypertension  Untreated HIV | O13, I10-I13  B20-B24, Z21, R75, O98.7 or test | C02  None |
| Treated HIV | B20-B24, Z21, R75, O98.7 or test | J05AE, J05AF, J05AG, J05AJ and J05AR |

antiretroviral medication for HIV treatment (ATC codes)

**eTable 5. Sensitivity analysis: association between risk factors and PPD**

|  | aHR (95%CI) |
| --- | --- |
| Age 10-17 | 1.02 (0.73-1.43) |
| Age 25-29 | 1.21 (1.05-1.39 |
| Age 30-34 | 1.2 (1.04-1.39) |
| Age 35-40 | 1.2 (1.03-1.4) |
| Age 41+ | 1.22 (0.96-1.55) |
| HIV | 1.03 (0.91-1.17) |
| PCOS | 1.4 (1.11-1.76) |
| Endometriosis | 1.06 (0.9-1.24) |
| Gestational diabetes | 0.9 (0.69-1.17) |
| Gestational hypertension | 1.32 (1.04-1.67) |
| Hyperemesis gravidarum | 1.35 (1.13-1.6) |
| Complicated labor/fetal distress | 0.92 (0.78- 1.09) |
| Preterm delivery | 1.51 (1.34-1.7) |
| Perineal laceration | 0.84 (0.64-1.11) |
| Elective C-section | 1.06 (0.97-1.16) |
| Emergency C-section | 1.1 (0.98-1.23) |
| Preeclampsia | 1.03 (0.88-1.22) |
| Eclampsia | 1.2 (0.74-1.95) |
| Postpartum hemorrhage | 1.3 (0.91-1.85) |
| History of depression | 3.75 (3.39-4.16) |

aHR: adjusted Hazard ratios, CI: Confidence Intervals, PCOS=polycystic ovary syndrome

Cox regression model was adjusted for calendar year and self-identified population group. Reference group for age: 18-24 years.
